# RNA-Seq Analysis of Human Cumulus Cells Identifies Angiogenic Pathways Associated with Infertility

**DOI:** 10.3390/cells15080677

**Published:** 2026-04-11

**Authors:** Alejandro Baratas, Victoria Pérez-Quiroga, Rosario Planello, Mónica Aquilino, Magdalena Serrano, Moisés de la Casa, Yosu Franco-Iriarte, Rosa Roy

**Affiliations:** 1 Grupo REDA, Departamento de Biología (Genética), Facultad de Ciencias, Universidad Autónoma de Madrid, 28049 Madrid, Spain; 2 Grupo de Entomología Molecular, Biomarcadores y Estrés Ambiental, Facultad de Ciencias, Universidad Nacional de Educación a Distancia (UNED), 28040 Madrid, Spain; 3 División de Genética, Facultad Ciencias de la Salud, Universidad Rey Juan Carlos, 28922 Alcorcón, Spain; 4 Departamento de Mejora Genética Animal, Instituto Nacional de Investigación y Tecnología Agraria y Alimentaria (INIA-CSIC), Ctra. de La Coruña km 7,5, 28040 Madrid, Spain; 5 GINEFIV, Assisted Reproduction Centre, 28703 Madrid, Spain; 6 Hospital Ruber Internacional, 28034 Madrid, Spain

**Keywords:** angiogenesis, cumulus cells, RNA-seq, angiopoietins, infertility, biomarkers

## Abstract

**Highlights:**

**What are the main findings?**

- RNA-seq analysis of human cumulus cells identified angiogenesis-related pathways as a major source of transcriptomic differences between oocyte donors and infertile patients.
- Several genes involved in vascular regulation (*ANGPT1*, *ANGPT2*, *E2F7*, *ANKRD22*, *NRP2*, and *THBS1*) showed coordinated expression changes, suggesting altered angiogenic signaling within the cumulus–oocyte complex.
- Among the validated genes, *ANKRD22* and *E2F7* displayed statistically significant differences, supporting their potential relevance in the follicular microenvironment.

**What are the implications of the main findings?**

- These results suggest that angiogenesis-related pathways may contribute to the molecular differences associated with infertility at the follicular level.
- Angiogenesis-related genes in cumulus cells may represent candidate markers for future studies aimed at improving the non-invasive assessment of oocyte competence.
- The findings provide a framework for further research exploring the role of follicular vascular dynamics in reproductive outcomes and ART success.

**Abstract:**

Non-invasive assessment of oocyte quality remains a challenge in assisted reproductive technology (ART). Through their bidirectional communication with the gamete, cumulus cells (CCs) act as a functional mirror of oocyte competence; however, the specific angiogenic signature within this microenvironment is still poorly understood. In the present study, we performed RNA-seq on CCs from healthy oocyte donors and infertile patients, utilizing a multi-pipeline bioinformatic approach (STAR-Cufflinks, TopHat-HTSeq, and HISAT2-StringTie) to establish a high-confidence, exploratory transcriptomic profile. A set of 234 differentially expressed genes (DEGs) consistently identified across pipelines was obtained, with functional enrichment highlighting blood vessel morphogenesis and angiogenesis as primary drivers of transcriptomic divergence between groups. RT-qPCR validation in individual samples confirmed statistically significant differences for *ANKRD22* (upregulated) and *E2F7* (downregulated) in infertile patients, while other angiogenesis-related genes, including *ANGPT1*, *ANGPT2* and *THBS1*, showed consistent but non-significant expression trends, suggesting alterations in angiogenesis-related processes within the follicular microenvironment. These findings support the presence of coordinated angiogenesis-related alterations in cumulus cells and provide a basis for future studies exploring their potential relevance in oocyte competence and ART outcomes.

## 1. Introduction

Recent advances in assisted reproductive technology (ART) have leveraged transcriptomic profiling to identify non-invasive biomarkers of oocyte competence. In this context, RNA sequencing (RNA-seq) of granulosa cells (GCs) has emerged as a particularly powerful approach. GCs, which are routinely discarded during follicular aspiration, offer unique and ethically non-invasive access to the follicular microenvironment, enabling researchers to uncover molecular signatures associated with oocyte quality and embryo developmental potential without compromising the gamete itself [1].

Granulosa cells play multifaceted roles in oocyte development through various mechanisms, including paracrine communication [2], gap-junctional coupling [3], and the regulation of key follicular processes. Their gene expression profiles reflect critical pathways such as cell cycle regulation, apoptosis, extracellular matrix remodelling, and notably, angiogenesis—all of which collectively dictate oocyte quality [4]. Accordingly, GCs are considered valuable indicators of follicular health and oocyte developmental competence.

While several transcriptomic studies of granulosa cells in both human [5,6] and animal models [7,8,9] have identified key gene signatures associated with reproductive outcomes, recent advances have further refined this landscape. For instance, Cadenas et al. [10] and Gan et al. [11] have identified specific transcriptomic fingerprints in CCs that serve as reliable predictors of oocyte developmental competence and clinical pregnancy success. However, a significant knowledge gap remains. Relatively few studies have specifically examined angiogenesis-related genes and their potential role in oocyte development and female infertility [12]. This gap is notable given the physiological importance of angiogenesis in ovarian function.

Angiogenesis—the formation of new blood vessels from existing ones—is a tightly regulated process that exhibits exceptional dynamism within the female reproductive system [13]. Unlike most adult tissues where angiogenesis occurs infrequently, the ovaries undergo cyclical waves of vascular growth and regression that are essential for follicular development, ovulation, and corpus luteum formation [14]. As follicles progress from the early to late stages of development, they transition from relying on passive diffusion [13] to requiring active neovascularization to meet their increasing metabolic and structural demands [15,16]. The extent of follicular vascularization not only influences follicular health, with insufficient angiogenesis being associated with follicular atresia [17], but also appears to contribute to the selection of the dominant follicle [18]. Thus, controlled angiogenesis emerges as a fundamental determinant of ovarian function and reproductive success.

Despite its biological relevance and the growing interest in GC transcriptomics, angiogenesis-related pathways remain underexplored in RNA-seq studies of human granulosa cells. To address this gap, we performed a comparative transcriptomic analysis of GCs from two clinically distinct groups: proven-fertile oocyte donors and infertile women undergoing ART.

The objectives of this study were twofold: first, to characterize global gene expression differences between fertile and infertile women; and second, to evaluate the differential expression of angiogenesis-related genes between these groups.

By investigating angiogenesis-related transcriptional patterns in GCs, this research aims not only to advance our understanding of how the granulosa cell transcriptome reflects the vascular dynamics of the ovarian follicle, but also to provide a basis for future development of clinically relevant tools in ART.

As suggested by recent profiling studies [10,19], the CC transcriptome is highly sensitive to the functional state of the oocyte. This study reinforces the potential of these cells as non-invasive diagnostic tools, specifically by characterizing how a suboptimal angiogenic niche may underline the reduced reproductive potential in infertile cohorts. Specifically, angiogenesis-related gene expression patterns may represent potential candidates for non-invasive assessment of oocyte quality, with possible implications for clinical decision-making, such as oocyte selection for vitrification or fertilization. such as oocyte selection for vitrification or fertilization. Ultimately, this work seeks to contribute to the development of more personalized and effective strategies in reproductive medicine.

## 2. Materials and Methods

### 2.1. Study Population and Ethical Statement

The study included a cohort of 30 women undergoing controlled ovarian stimulation for in vitro fertilization (IVF) at GINEFIV Assisted Reproduction Centre in Madrid. Participants were stratified into two groups: Oocyte donors (*n* = 15) (30.9 ± 3.7 years) and infertile women undergoing IVF due to unexplained infertility (*n* = 15) (36.3 ± 5.1 years).

The study protocol was approved by the Ethics Committee of the Autonomous University of Madrid (CEI-106-2075) and conducted following the Declaration of Helsinki. All participants provided written informed consent.

### 2.2. Inclusion and Exclusion Criteria

All women included in the study were clinically healthy, with a normal body mass index and no known metabolic, endocrine, or gynecological disorders. For the infertile group, patients were included based on the failure to achieve pregnancy after at least 12 months of unprotected intercourse, without a specific diagnosis of female factor infertility (unexplained infertility). All participants reported no unhealthy lifestyle habits, including smoking or excessive alcohol consumption.

### 2.3. Sample Collection and Processing

Women received treatment to induce multiple follicle development, with either ovarian stimulation with GnRH agonist long protocol or antagonist short protocol and using recombinant follicle-stimulating hormone (FSH) or human menopausal gonadotropin (hMG). Stimulation was monitored using the ultrasound measurement of follicle number and diameter. When at least two follicles presented a diameter greater than 17 mm, ovulation was induced with 250 µg of recombinant human chorionic gonadotrophin (rhCG). Transvaginal ultrasound-guided follicular puncture was performed 36 h after rhCG injection.

Cumulus cells (CCs) were retrieved mechanically during oocyte denudation at the time of oocyte collection. Immediately following recovery, CCs were preserved in 1 mL of TRIzol reagent (15596018, Life Technologies, Carlsbad, CA, USA) and stored at −80 °C for subsequent molecular analysis.

### 2.4. RNA Purification

Total RNA was purified using the PureLink™ RNA Mini Kit (Invitrogen, Carlsbad, CA, USA) following the manufacturer’s instructions. RNA was eluted in 60 µL of RNase-free water. RNA concentration and purity were determined using a NanoDrop™ 1000 spectrophotometer (Thermo Scientific, Braunschweig, Germany), and RNA integrity was assessed with an Agilent 2100 Bioanalyzer using the RNA 6000 Pico Chip (5067-1513, Agilent Technologies, Santa Clara, CA, USA). RNA integrity numbers (RINs) were within the acceptable range for downstream transcriptomic analyses and are provided in the Supplementary Material.

### 2.5. Library Preparation and Sequencing

Due to the limited amount of RNA obtained from individual cumulus cell samples and the exploratory nature of the study, RNA samples were pooled prior to library preparation to ensure sufficient RNA input. Pools were generated by combining equal amounts of high-quality RNA from each individual to ensure balanced contribution and minimize bias. Each pool therefore represents the average transcriptomic profile of the corresponding group. Each pool was generated by combining equal amounts of RNA from 3 individuals, a strategy commonly used in exploratory RNA-seq studies to ensure sufficient RNA input while maintaining biological representativeness [20].

RNA library preparation and sequencing were conducted at the Genomics Unit, Madrid Science Park, Cantoblanco Campus (Madrid, Spain). For each pool, 500 ng of RNA was used to generate libraries with the Illumina TruSeq Stranded mRNA Sample Preparation Kit (Illumina, San Diego, CA, USA). Single-end sequencing (1 × 76 bp) was conducted on a NextSeq500 sequencer (Illumina, San Diego, CA, USA).

### 2.6. Bioinformatic Analysis

To ensure robustness of differential expression analysis, multiple independent bioinformatic pipelines were applied. Only differentially expressed genes (DEGs) identified by at least two pipelines were considered for downstream analysis.

Read quality was assessed using FastQC (v0.11.9) [21]. Illumina adapters were removed using Trimmomatic (v0.39) [22] with default parameters. Trimmed reads were aligned to the *Homo sapiens* reference genome (GRCh38.p13) [23], and analysed using the following three pipelines:

1. STAR-Cufflinks Pipeline: Reads were aligned using STAR (v2.7.10a) [24] with default settings. Gene quantification was performed with Cufflinks (v2.2.1) [25], followed by normalization and differential expression analysis using Cuffdiff (v2.2.1) [25].
2. TopHat-HTSeq-DESeq2 Pipeline: Reads were aligned using TopHat (v2.2.1) [26], quantified with HTSeq (v0.13.5) [27], and analysed for differential expression using DESeq2 (v1.36.0) [28].
3. HISAT2-StringTie-DESeq2 Pipeline: Reads were aligned using HISAT2 (v2.2.1) [29], assembled with StringTie (v2.2.1) [30], and analysed with DESeq2 (v1.36.0) [28].

Differentially expressed genes (DEGs) were identified using a Benjamini–Hochberg false discovery rate (FDR)-adjusted *p*-value threshold of <0.05. DEGs common to at least two pipelines were subjected to enrichment analysis using Gene Ontology (GO; release 2023) [31,32] and STRING (v12.0) [33]. The top 20 enriched GO terms were visualized using RStudio with R (v2023.09.1) [34]. Key findings were subsequently validated by RT-qPCR using individual samples.

### 2.7. RT-qPCR 

To provide independent biological validation of the transcriptomic findings, RT-qPCR was performed using a separate cohort of individual samples (*n* = 9 oocyte donors and *n* = 9 infertile patients) that were not included in the original sequencing pools.

Ten genes were selected based on their involvement in blood vessel development, biological significance in CCs, and fold-change. For each sample, RNA (0.5 µg) was reverse-transcribed to cDNA using the iScript Advanced cDNA Synthesis Kit (BioRad Laboratories, Hercules, CA, USA) in a 20 µL reaction containing 4 µL 5× iScript Advanced Reaction Mix, 1 µL iScript Advanced Reverse Transcriptase, and nuclease-free water. Reverse transcription was performed at 46 °C for 20 min, followed by 95 °C for 1 min.

Quantitative PCR (qPCR) was performed in duplicate using a QuantStudio 1 Real-Time PCR System (Applied Biosystems, Waltham, MA, USA). Each 10 µL reaction contained 5 µL PowerUp SYBR Green Master Mix (A25742, Applied Biosystems, Vilnius, Lithuania), 2 µL cDNA, 1 µL primer pair (10 µM each), and 2 µL nuclease-free water. (Primer sequences are listed in Supplementary Table S1). *GAPDH* and *B2M* were used as housekeeping genes, and their expression stability was verified using geNorm. Cycling conditions were: 50 °C for 2 min, 95 °C for 2 min, followed by 40 cycles of 95 °C for 15 s and 56 °C for 30 s. A melt curve analysis (95 °C for 15 s, 60 °C for 1 min, 95 °C for 15 s) confirmed primer specificity.

Gene expression was quantified using the ΔΔCt method [35]. Ct values were normalized to the geometric mean of *GAPDH* and *B2M*. Normalised Ct values considered outliers (values < Q1 − 1.5 × IQR and values > Q3 + 1.5 × IQR) were excluded from the analysis. Valid normalised Ct values were relativised to the control average and represented in a box-and-whisker plot using RStudio. Significant differences (*p*-value < 0.05) were determined using the Mann–Whitney U test in SPSS (IBM, Armonk, NY, USA).

## 3. Results

### 3.1. Transcriptomic Profiling of Cumulus Cells

Cumulus cells (CCs) were collected from six oocyte donors and six women undergoing in vitro fertilisation (IVF) and each group was pooled into two biological replicates (Pool_D1 and Pool_D2 for oocyte donors; Pool_PM1 and Pool_PM2 for IVF patients) to enable RNA-seq analysis. This pooling strategy was implemented to obtain sufficient RNA input and is consistent with exploratory transcriptomic approaches aimed at identifying robust group-level expression patterns. Unsupervised hierarchical clustering and principal component analysis (PCA) were performed to assess transcriptomic relationships between samples.

**Figure 1:**
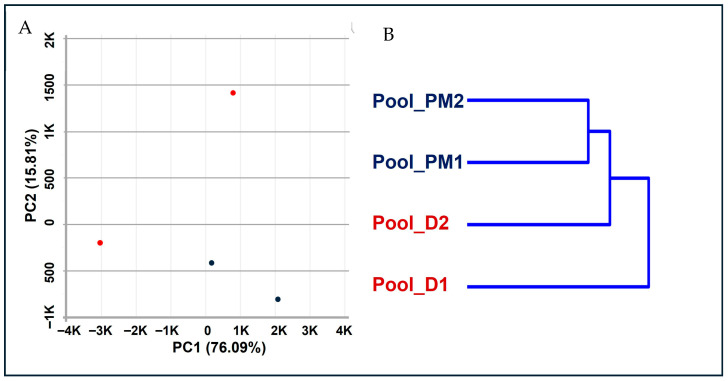
Global transcriptomic analysis of cumulus cells. (**A**) Principal component analysis (PCA) showing separation between oocyte donor (red) and IVF patient (blue) transcriptomes, with the first two components explaining 91.9% of the total variance. (**B**) Unsupervised hierarchical clustering of samples based on gene expression profiles showing grouping according to clinical group, with donor pools (Pool_D1, Pool_D2) and patient pools (Pool_PM1, Pool_PM2) forming distinct clusters.

Hierarchical clustering revealed greater grouping by clinical category, with oocyte donor pools (Pool_D1 and Pool_D2) clustering together, and IVF patient pools (Pool_PM1 and Pool_PM2) (Figure 1B) forming a separate cluster. PCA showed that the first two principal components explained 91.9% of the total variance (PC1: 76.09%, PC2: 15.81%), indicating that the major sources of variability in the dataset were effectively captured (Figure 1A). Principal component analysis demonstrated clear transcriptomic differences between donors and IVF patients, consistent with distinct transcriptomic profiles. Although some intra-group variability was observed, it was more pronounced among donor pools, whereas patient samples exhibited comparatively tighter clustering.

### 3.2. Differential Gene Expression Analysis

To account for the limited number of pooled replicates, differential expression analysis was conducted three independent bioinformatic pipelines (see Section 2). This multi-pipeline strategy was adopted to increase robustness and reduce method-specific bias in DEG identification.

**Figure 2:**
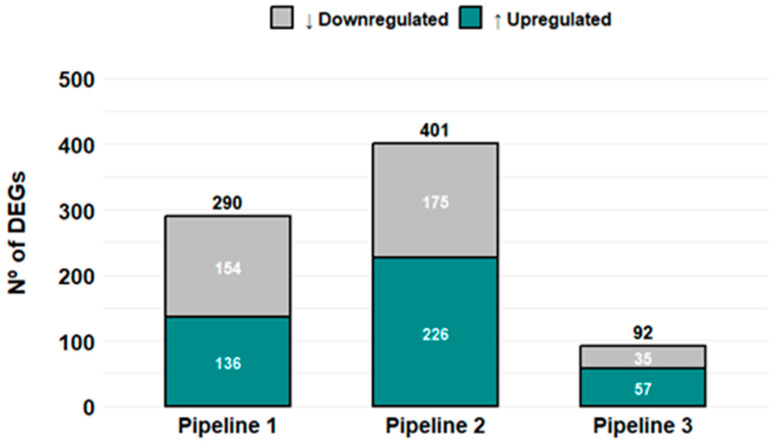
Pipeline-specific detection of differentially expressed genes. Bar plot showing the number of upregulated (↑) and downregulated (↓) DEGs identified by each of the three bioinformatic pipelines comparing oocyte donors and infertile patients. Pipeline 2 detected the highest number of DEGs (*n* = 401), followed by Pipeline 1 (*n* = 290) and Pipeline 3 (*n* = 92). DEGs were defined according to the statistical thresholds specified in the Section 2.

All pipelines achieved >90% read alignment to the *Homo sapiens* reference genome (hg38) (Supplementary Table S2). The number of differentially expressed genes (DEGs, adjusted *p* < 0.05) varied across pipelines: Pipeline 2 identified 401 DEGs (56% upregulated), Pipeline 1 identified 290 DEGs (46% upregulated), and Pipeline 3 identified 92 DEGs (61% upregulated) (Figure 2).

**Figure 3:**
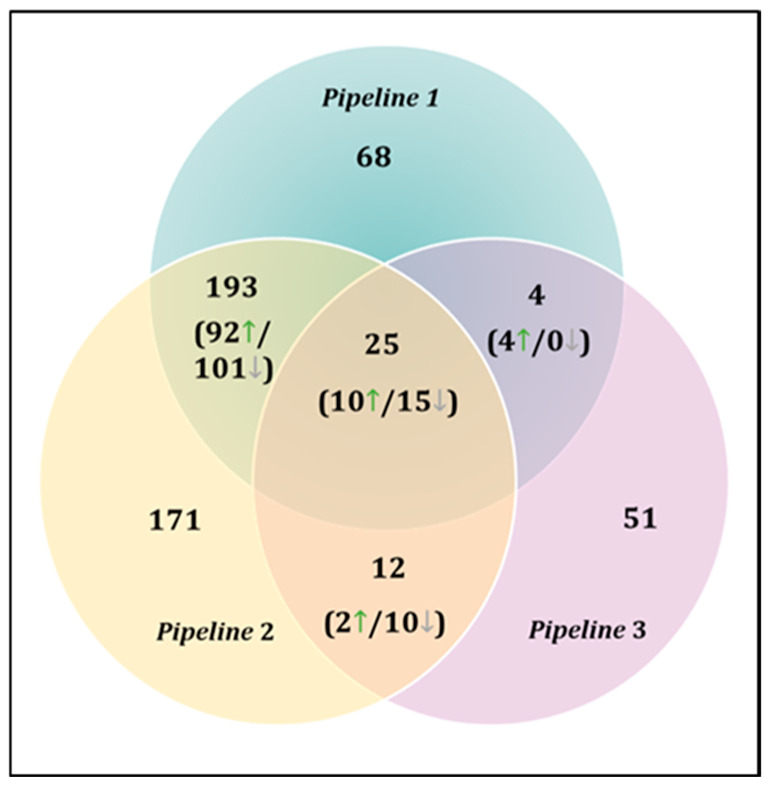
Overlap of differentially expressed genes across bioinformatic pipelines. Venn diagram illustrating the consensus of 234 DEGs identified in at least two pipelines, with 25 genes common to all three. The highest concordance was observed between Pipeline 1 and 2 (193 shared DEGs). Numbers in parentheses indicate the distribution of upregulated and downregulated genes within each overlap. Green arrows (↑) denote upregulated genes, whereas grey arrows (↓) indicate downregulated genes in infertile patients relative to oocyte donors.

To enhance confidence in DEG selection, only genes identified in at least two pipelines were retained for downstream analyses. This consensus approach yielded 234 DEGs, including 25 genes consistently detected across all three pipelines (Figure 3).

The highest overlap was observed between Pipelines 1 and 2 (193 shared DEGs), while smaller overlaps were detected between Pipelines 1 and 3 (4 DEGs) and Pipelines 2 and 3 (12 DEGs). This strategy prioritizes reproducible transcriptional signals and is particularly relevant in exploratory datasets with limited biological replication.

### 3.3. Functional Enrichment Analysis

**Figure 4:**
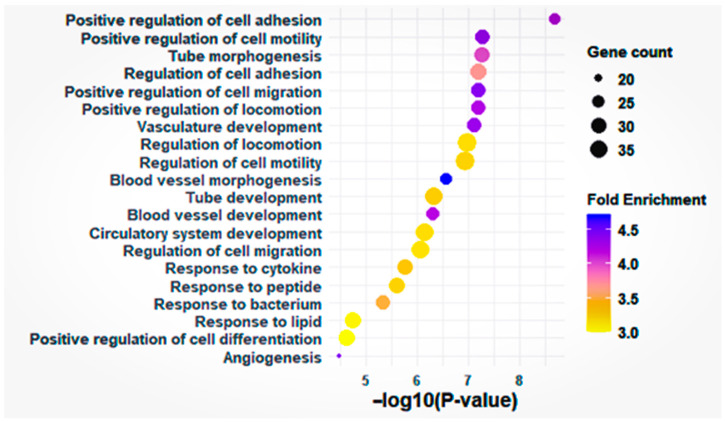
Enrichment analysis of Gene Ontology biological processes. Scatter plot of the top 20 significantly enriched GO terms among the 234 consensus DEGs. Point size refers to the number of genes per term, color indicates fold enrichment (purple: high, yellow: low). Enriched terms include processes related to angiogenesis, blood vessel development, and cell migration.

Gene Ontology (GO) analysis of the 234 DEGs revealed significant enrichment in biological processes related to cell motility, vasculature development, and responses to external stimuli (Figure 4). The most significant pathways were associated with cell migration, including *Positive regulation of cell motility* (GO:2000147; *p* = 5.22 × 10^−8^, fold enrichment = 4.35, 29 DEGs), *Positive regulation of cell migration* (GO:0030335; *p* = 6.32 × 10^−8^, fold enrichment = 4.40, 28 DEGs), and “Regulation of locomotion” (GO:0040012; *p* = 1.04 × 10^−7^, fold enrichment = 3.20, 39 DEGs) (Figure 4). Pathways related to vasculature development showed the highest fold enrichment, including *Blood vessel morphogenesis* (GO:0048514; *p* = 2.76 × 10^−7^, fold enrichment = 4.72, 24 DEGs) and *Angiogenesis* (GO:0001525; *p* = 3.19 × 10^−5^, fold enrichment = 4.45, 18 DEGs).

**Figure 5:**
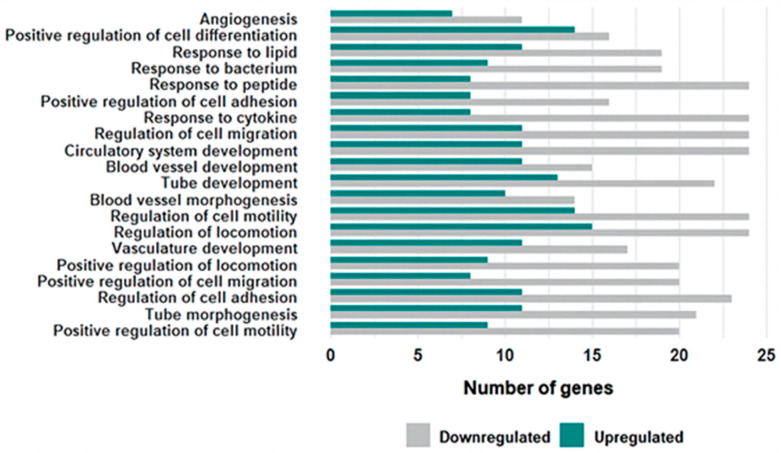
Directional enrichment of biological pathways. Bar plot showing the 20 most significantly enriched GO biological pathways comparing infertile patients and oocyte donors. Gray bars represent downregulated genes in patients; green bars represent upregulated genes in patients. Several angiogenesis-related terms are predominantly associated with genes downregulated in patients.

Notably, downregulated genes predominated in the top 20 enriched GO terms (Figure 5). The genes involved in the 20 pathways are shown in Supplementary Table S3. Focus on angiogenesis-related pathways, *ANGPT1* and *CALCRL* were upregulated, while *E2F7*, *NRP2*, *NRXN3*, and *THBS1* were downregulated in IVF patients compared to oocyte donors (Supplementary Table S3).

Complementary network analysis using the STRING platform, based on the 234 DEGs mentioned above, identified a large core involving genes related to angiogenesis (e.g., *ANGPT1*, *THBS1*, *BMP4*, *GLUL*) and endothelial-associated factors (e.g., *VCAM1*, *ICAM1*, *LIF*) (Supplementary Figure S1).

### 3.4. qPCR Validation

**Figure 6:**
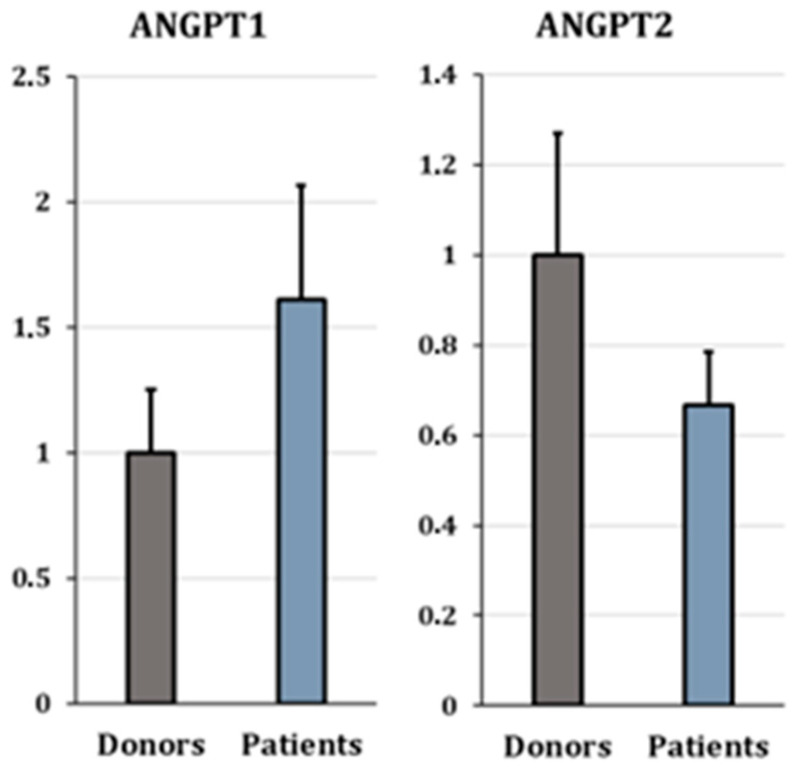
Expression analysis of *ANGPT1* and *ANGPT2*. Bar plots showing relative mRNA levels (ΔΔCt method) of *ANGPT1* and *ANGPT2* in cumulus cells from oocyte donors (gray) and infertile patients (blue). Bar plots showing mean ± SEM (Standard Error of the Mean). Expression levels were normalized to the selected reference gene(s) as described in the Section 2.

**Figure 7:**
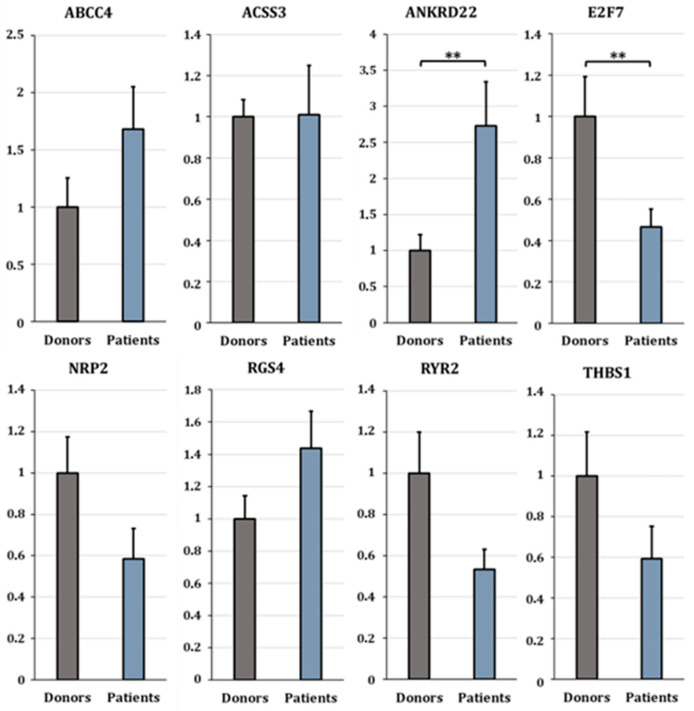
RT-qPCR validation of selected differentially expressed genes. Boxplots showing relative expression levels (ΔΔCt) of *ABCC4*, *ACSS3*, *ANKRD22*, *E2F7*, *NRP2*, *RGS4*, *RYR2*, and *THBS1* in cumulus cells from oocyte donors (grey) and infertile patients (blue) cumulus cells. Asterisks indicate statistically significant differences (** *p* < 0.05). Only *ANKRD22* and *E2F7* reached statistical significance, while the remaining genes showed consistent but non-significant expression trends.

To validate the transcriptomic findings, ten differentially expressed genes (DEGs) were selected for RT-qPCR analysis based on three criteria: (1) involvement in angiogenesis (*ANGPT1*, *E2F7*, *NRP2*, *THBS1*), (2) established relevance in cumulus cell biology (*ABCC4*, *ANKRD22*, *RYR2*), and (3) strong and consistent differential expression across pipelines (fold-change > 2; *RGS4*, *ACSS3*). Additionally, *ANGPT2*—a known regulator of vascular stability—was included, although it was only detected in Pipeline 2 (Figure 6 and Figure 7 and Table 1).

Validation was performed in an independent cohort of individual samples, providing complementary evidence at the biological level. RT-qPCR analysis confirmed the direction of change for 9 out of 10 genes, with *RGS4* being the only gene showing a discordant trend relative to RNA-seq results (Table 1). Differences in fold-change magnitude between RNA-seq and RT-qPCR are expected and may reflect methodological differences in normalization, sensitivity, and the use of pooled versus individual samples.

The expression of *ANGPT1* (Figure 6) was slightly higher in patients compared to donors, though this difference was not statistically significant (log2 fold change = 0.69, *p* > 0.05). In contrast, *ANGPT2* (Figure 6) expression was slightly lower in patients (fold-change = −0.58, *p* > 0.05).

Among other angiogenesis-related genes, *ANKRD22* was significantly upregulated in patients (log2 fold change = 1.45, *p* = 0.033), while *E2F7* was significantly downregulated (log2 fold change = −1.04, *p* = 0.024) (Figure 7). *NRP2* and *THBS1* were also downregulated in patients, consistent with the RNA-seq results, though these differences did not reach statistical significance in the qPCR cohort.

Expression trends for the remaining genes (*ABCC4*, *ACSS3*, *RYR2*) were also consistent with RNA-seq data, albeit without statistical significance. Only *RGS4* displayed a discordant expression pattern, showing slight upregulation by qPCR despite being downregulated in all three RNA-seq pipelines (Figure 7 and Table 1).

Overall, the concordance in expression trends between RNA-seq and qPCR for most genes (9/10) supports the consistency of the observed angiogenesis-related transcriptional patterns.

## 4. Discussion

The cellular transcriptome serves as a direct reflection of a cell’s functionality and physiological state. In the context of human reproduction, and more specifically in fertilization through assisted reproductive technologies (ART), the interaction between the two gametes—the spermatozoon and the oocyte—is paramount. However, the molecular study of the oocyte itself presents an unavoidable practical limitation: the analysis of its transcriptome necessitates its destruction, thereby precluding any subsequent assessment of its developmental competence. To circumvent this obstacle, our investigation focused on the cumulus cells (CCs) that intimately surround the oocyte, and form a tightly a coordinated functional unit with it [36].

To explore the female contribution to reproductive outcomes, we compared the transcriptome profile of CCs from two well-defined cohorts: oocyte donors (with proven fertility) and infertile patients undergoing ART. This approach provides a framework to investigate transcriptomic differences potentially associated with oocyte quality and the follicular microenvironment, although causality cannot be directly inferred. 

### 4.1. Global Transcriptomic Landscape Reveals Conserved Identity with Specific Dysregulation in Infertile Patients

Our initial global transcriptomic profiling yielded important insights. Principal component analysis (PCA) showed that the first two components explained 91.9% of the total variance, with separation between donor and patient CCs primarily captured along the main axis of variation. This suggests the presence of measurable inter-group transcriptomic differences, while preserving the shared cellular identity of cumulus cells. Consistently, unsupervised hierarchical clustering demonstrated grouping by clinical category, supporting the presence of group-specific gene expression patterns. RNA-seq was performed on two pools of three donors and two pools of three infertile patients, an approach frequently used in exploratory transcriptomic studies when biological material is limited and the aim is to identify robust group-level signals [20,37]. While this pooling strategy limits the assessment of inter-individual variability and statistical power, it is suitable for exploratory analyses aimed at identifying consistent transcriptional trends.

It is also worth noting that the age difference between donors and patients could have contributed to some of the observed transcriptional variation, as age has been associated with changes in follicular gene expression. However, the clear clustering by clinical group suggests that the main transcriptomic patterns identified are primarily linked to reproductive status.

Together, these findings suggest that infertility-associated molecular alterations may occur within a conserved cellular framework rather than reflecting a complete transcriptional reprogramming. This observation is consistent with previous transcriptomic studies of granulosa and cumulus cells, which have shown that oocyte competence is often associated with subtle pathway-specific alterations rather than large-scale transcriptomic changes [38].

### 4.2. Angiogenesis and Vascular Development Pathways Are Differentially Regulated in Infertile Patients

To identify transcriptomic differences between groups, we performed RNA-seq and analysed differential expression using three independent bioinformatic pipelines. This robust, multi-method approach identified a set of 234 differentially expressed genes (DEGs) common to at least two pipelines—including 25 DEGs shared across all three. Given the limited number of pooled samples, this consensus-based strategy was used to prioritize genes showing consistent signals across analytical methods rather than relying on single-pipeline outputs.

The variation in the number of DEGs identified across pipelines likely reflects methodological differences at multiple analytical stages. At the alignment level, STAR [24], HISAT2 [29], and TopHat [26] employ distinct splice-aware algorithms and strategies for handling multi-mapped reads and junction detection, which can subtly influence the composition of gene-level count matrices. During quantification, HTSeq [27] performs strict gene-level counting, whereas StringTie [30] reconstructs transcripts and redistributes reads across isoforms, and Cufflinks [25] estimates expression using FPKM normalization; these approaches differ in how ambiguously assigned reads and low-abundance transcripts are modelled. Finally, downstream statistical analysis introduces further variability: DESeq2 [28] applies a negative binomial framework with gene-specific dispersion estimation and log2 fold-change shrinkage, while Cuffdiff [25] relies on alternative variance modelling and normalization strategies. These methodological differences may influence significance thresholds, particularly in datasets with limited biological replication. Thus, even when starting from identical raw sequencing reads, alternative bioinformatic processing strategies can yield variable DEG counts, underscoring the importance of consensus-based approaches to identify the most robust and reproducible transcriptional signals.

Functional characterization of these 234 consensus DEGs using Gene Ontology enrichment and STRING network analysis indicated that the transcriptomic divergence between donors and patients were enriched in pathways related to angiogenesis, cell migration, and vascular development.

Specifically, we observed significant enrichment in terms such as *Blood vessel morphogenesis* (GO:0048514), *Angiogenesis* (GO:0001525), and *Positive regulation of cell migration* (GO:0030335). Functional enrichment analysis of these consensuses differentially expressed genes revealed a striking overrepresentation of biological processes related to vascular remodeling and endothelial dynamics.t. These observations are consistent with the known role of follicular vascularization in supporting oocyte growth, steroidogenesis, and metabolic exchange [39].

To further explore these findings, a subset of 10 genes was selected for qPCR validation based on their consistent identification across all three RNA-seq pipelines (Table 1) and their involvement in complementary aspects of angiogenesis (Table 1). Importantly, validation was performed using individual samples not included in the RNA-seq pools, providing additional biological support for the observed expression patterns and partially addressing limitations associated with pooling strategies [40].

RT-qPCR analysis confirmed the direction of expression changes for most genes, although only a subset reached statistical significance. Specifically, *ANKRD22* and *E2F7* showed statistically significant differences between groups, whereas other genes displayed consistent but non-significant trends. Differences in fold-change magnitude between RNA-seq and qPCR are expected and may reflect differences in normalization methods, sensitivity, and the use of pooled versus individual samples. Importantly, the consistent direction of change across platforms supports the presence of consistent expression patterns across platforms.

### 4.3. Dysregulation of the Angiopoietin–Tie Axis and Other Genes Involved in Vascular Remodeling

This study integrates RNA-seq discovery with independent RT-qPCR validation to characterize angiogenesis-related transcriptional alterations in CCs from oocyte donors and infertile women. Together, these analyses suggest that infertility may be associated with a coordinated dysregulation of angiogenic signaling, affecting both the angiopoietin–*Tie* axis and multiple downstream genes involved in vascular remodeling, endothelial signaling, metabolic adaptation, and follicular microenvironment homeostasis.

The combined results suggest that infertility is not associated with a global loss of cumulus cell identity, but rather with more subtle alterations in pathways regulating vascular plasticity and follicular support. Among the analyzed genes, *ANKRD22* and *E2F7* showed statistically significant differences between donors and patients, while *ANGPT1* and *ANGPT2*, together with *NRP2*, *THBS1*, *ABCC4*, *RYR2*, *ACSS3*, and *RGS4*, provided additional context through consistent but non-significant expression patterns (Table 2).

#### 4.3.1. Dysregulation of the ANGPT–Tie Axis in Patients

*ANGPT1* and *ANGPT2* are central regulators of ovarian angiogenesis, acting through the Tie2 receptor to balance vascular stability and remodeling. *ANGPT1* promotes vessel maturation and stabilization, whereas *ANGPT2* facilitates vascular plasticity by loosening endothelial–pericyte interactions and sensitizing vessels to *VEGF* signaling [51,52].

In our study, *ANGPT1* expression showed a trend toward higher levels in infertile patients, while *ANGPT2* was relatively higher in donors. This pattern is consistent with previous observations showing that *ANGPT2* predominates in larger, developmentally competent follicles, whereas *ANGPT1* is associated with smaller or less mature follicles [53,54].

The significance of *ANGPT2* as a marker of follicular progression is further supported by Cadenas et al. [10], who identified it as one of the most highly upregulated genes (over 100-fold increase) during the transition from immature germinal vesicle (GV) oocytes to mature metaphase II (MII) stages. Their findings highlight *ANGPT2* as a critical transcriptomic signature of oocyte maturation and developmental competence.

Because CCs in this study were collected from pre-ovulatory follicles, the observed shift toward a more destabilized angiogenic profile in infertile patients may reflect premature or dysregulated vascular remodeling. Such alterations could potentially influence oxygen and nutrient delivery to the oocyte, thereby compromising developmental competence [13].

#### 4.3.2. *E2F7* and *ANKRD22*: Transcriptional and Stress-Related Regulators

Among the validated genes, *E2F7* and *ANKRD22* showed the most consistent and biologically meaningful differences between donors and patients.

E2F7 was significantly downregulated in infertile patients. Members of the E2F family are key regulators of cell-cycle progression, proliferation, and apoptosis, and have also been implicated in endothelial cell function and angiogenic signaling [55,56]. In particular, E2F7 has been shown to regulate *VEGF*-responsive pathways and endothelial proliferation [46]. Reduced E2F7 expression may suggest reduced proliferative and angiogenic capacity in cumulus cells, potentially affecting follicular support functions.

In contrast, *ANKRD22* was significantly upregulated in infertile patients. Although this gene has been primarily studied in oncological contexts, recent evidence indicates that *ANKRD22* is responsive to microenvironmental stress and participates in cellular adaptation to altered metabolic and immune changes [57]. Its increased expression in infertile CCs may therefore an association with altered follicular conditions, possibly linked to changes in vascular or metabolic homeostasis.

Taken together, these findings are consistent with a potential shift toward reduced proliferative activity and altered stress-related signaling in infertile follicles. However, given the exploratory nature of the study, these interpretations should be considered preliminaries.

#### 4.3.3. Additional Angiogenesis-Related Genes

Several additional genes analyzed in this study provide further context for the angiogenesis-related alterations observed. Among them, *NRP2*, a *VEGF* co-receptor involved in endothelial guidance and vascular patterning, was downregulated in patients, suggesting potentially reduced responsiveness to *VEGF*-driven angiogenic cues and impaired vascular plasticity within the follicle [58]. In parallel, *THBS1*, an endogenous inhibitor of angiogenesis that contributes to vascular stabilization and homeostasis, showed higher expression in donors. Given its role in restraining excessive angiogenic activation [52,59], reduced *THBS1* levels in patients may be consistent with a less tightly regulated vascular environment.

In addition to these vascular regulators, genes involved in intracellular signaling and follicular communication were also altered. *RYR2*, which mediates intracellular Ca^2+^ release, was more highly expressed in donors. Calcium signaling plays a central role in cumulus–oocyte communication and oocyte maturation, and *RYR2* expression has previously been associated with oocyte competence and coordinated follicular signaling [60]. Its reduced expression in infertile patients may be associated with altered signaling dynamics in infertile follicles. *ABCC4*, which was more highly expressed in infertile patients, encodes a transporter involved in the efflux of prostaglandin E2 (*PGE2*), a key mediator of cumulus expansion, angiogenesis, and ovulatory processes. Altered *ABCC4* expression may be associated which dysregulated prostaglandin within the follicle, potentially contributing to abnormal paracrine signaling between granulosa cells, endothelial cells, and the oocyte [61,62]. Although *ABCC4* is not a primary angiogenic factor, its upregulation in patients supports the concept of disrupted communication between metabolic and vascular pathways in the infertile follicular environment.

Finally, *ACSS3* and *RGS4*, while included among the validated genes, showed limited discriminatory value. *ACSS3* exhibited similar expression levels in donors and patients, suggesting that acetate-dependent metabolic pathways are not major drivers of the observed phenotype. *RGS4* displayed inconsistent trends between RNA-seq and qPCR analyses, indicating a context-dependent or secondary role. Nevertheless, given its known inhibitory effects on *VEGF*-mediated signaling and endothelial tubulogenesis [63], its inclusion could supports the presence of multilayered regulation of angiogenic signaling in cumulus cells.

Taken together, these genes suggest the involvement of a coordinated network in which angiogenic signaling, vascular regulation, and intracellular communication contribute to the follicular microenvironment. The consistency of expression trends across multiple genes supports a pathway-level alteration, despite limited statistical significance at the individual gene level.

### 4.4. Integrated Interpretation and Clinical Implications

Collectively, these findings support a model in which fertile follicles maintain a finely regulated angiogenic balance characterized by appropriate vascular plasticity, controlled endothelial activation, and coordinated metabolic support. In contrast, infertile follicles may display a transcriptional profile suggestive of excessive vascular stabilization, reduced angiogenic responsiveness, and altered stress adaptation.

This molecular divergence may have direct implications for ART outcomes, as a suboptimal vascular network is inherently linked to impaired follicular oxygenation. Reduced oxygen tension and the resulting oxidative stress are known to disrupt oocyte mitochondrial biogenetics and redox balance, as well as induce oxidative DNA damage. These factors impair oocyte in vitro maturation and embryo development, leading to poor pregnancy out- comes in IVF [64]

**Figure 8:**
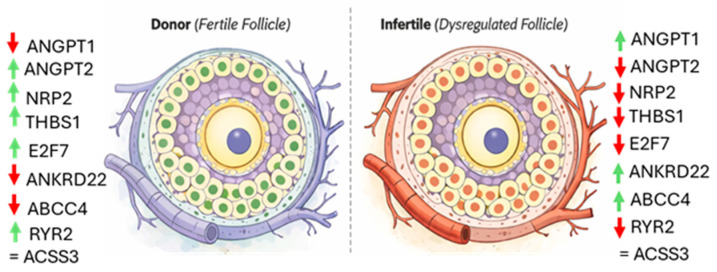
Schematic representation of angiogenesis-related gene expression patterns in cumulus cells. Diagram illustrates relative gene expression differences in cumulus cells from oocyte donors and infertile patients. The schematic summarizes trends observed in RNA-seq and RT-qPCR analyses and is intended as a conceptual representation. Green arrows represent increased gene expression, whereas red arrows indicate decreased gene expression.

The coordinated dysregulation of *E2F7*, *ANKRD22*, *NRP2*, and *THBS1* suggests that impaired angiogenic signaling could represent a central molecular feature of infertility at the follicular level. Rather than isolated defects, these alterations likely reflect a systemic failure in the ‘angiogenic switch’ required for proper follicular maturation. In a clinical context, such a compromised microenvironment may explain why some patients, despite producing morphologically ‘normal’ oocytes, experience poor blastocyst development or repeated implantation failure [65]. Importantly, these alterations were detected in cumulus cells, which are accessible during ART procedures and reflect the functional state of the oocyte microenvironment (Figure 8).

These angiogenesis-related genes may represent potential candidates for future biomarker development. By quantifying the expression of these vascular regulators, clinicians could move beyond morphology-based selection towards a more objective, molecular-based assessment of developmental potential.

Further studies are required to determine whether these markers could support decision-making] in assisted reproduction, including oocyte selection and treatment optimization. Validation in larger, independent, and age-matched cohorts will be necessary before clinical application.

#### Limitations and Future Directions

This study provides a transcriptomic characterization of cumulus cells from oocyte donors and infertile patients, identifying angiogenesis-related pathways as distinguishing features between groups. While the sample size was limited, the use of pooled samples enabled the generation of high-quality RNA-seq data with sufficient depth to capture consistent group-level transcriptional patterns, albeit at the expense of inter-individual resolution.

To complement this approach, selected genes were evaluated by RT-qPCR in an independent cohort of individual samples. This analysis confirmed the directionality of expression changes for most genes, although only a subset reached statistical significance. These findings support the interpretation of the RNA-seq data primarily at the pathway level and underscore the exploratory nature of the transcriptomic analysis.

Pooling strategies are widely employed in exploratory RNA-seq studies when biological material is limited and are particularly useful for identifying reproducible molecular signatures across groups [49]. In this context, the convergence between RNA-seq trends and RT-qPCR validation reinforces the biological consistency of the angiogenesis-related alterations observed.

Future studies should aim to extend these findings by incorporating larger cohorts and ideally age-matched cohorts and higher-resolution approaches, such as individual-sample or single-cell transcriptomics, to better capture follicular heterogeneity and refine the identification of clinically relevant molecular signatures.

Taken together, the coherence of the angiogenesis-related pathways identified, combined with the cross-platform consistency of expression trends, provides a strong conceptual framework for further investigation into the role of follicular vascular dynamics in oocyte competence and its potential relevance in assisted reproductive technologies.

## 5. Conclusions

Our transcriptomic analysis of human cumulus cells identifies angiogenesis-related pathways as key molecular features distinguishing infertile patients from oocyte donors and underscores the relevance of the follicular microenvironment in supporting oocyte developmental competence. The observed expression patterns in genes such as *ANGPT1*, *ANGPT2*, *E2F7*, *ANKRD22*, *NRP2*, and *THBS1* suggest that alterations in angiogenic signaling may contribute to differences in follicular function. These findings provide novel insight into the molecular mechanisms linking follicular vascular dynamics with reproductive potential and highlight cumulus cells as a valuable, non-invasive source of molecular information.

From a clinical perspective, these angiogenesis-related genes may represent potential candidates for non-invasive assessment of oocyte quality and follicular competence. Given that cumulus cells are a direct window into the oocyte’s microenvironment and are readily accessible during ART procedures, further validation is required to determine their potential utility in clinical decision-making.

Ultimately, these results provide a foundation for future studies aimed at refining oocyte selection strategies and exploring the role of follicular angiogenesis in reproductive outcomes.

## Figures and Tables

**Table 1:** Differentially expressed genes (DEGs) were selected for RT-qPCR analysis based on their relation with angiogenesis. Genes shown in red indicate downregulated expression, whereas genes shown in green indicate upregulated expression.

Genes	Log2 Fold Change			
	Pipeline 1	Pipeline 2	Pipeline 3	qPCR
*ANGPT1*	2.133	2.165	1.944	0.688
*ANGPT2*	−1.895	−1.935	−1.958	−0.583
*ABCC4*	1.165	1.163	1.107	0.749
*ACSS3*	1.265	1.266	1.241	0.015
*ANKRD22*	2.121	2.102	2.011	1.449
*E2F7*	−1.417	−1.418	−1.419	−1.038
*NRP2*	−2.269	−2.175	−1.818	−0.772
*RGS4*	−2.232	−2.237	−2.233	0.522
*RYR2*	−1.461	−1.445	−1.514	−0.906
*THBS1*	−1.161	−1.151	−1.151	−0.755

**Table 2:** Differentially expressed genes (DEGs) were selected for RT-qPCR analysis based on their relationship with angiogenesis and vascular plasticity.

Gene	Main Molecular/Cellular Function (Simplified)	Relationship to Angiogenesis and Vascular Plasticity	Reference
*ANGPT1*	Secreted ligand of the Tie2 receptor that promotes endothelial cell survival, pericyte recruitment, and vascular stabilization.	Pro-angiogenic and vessel-stabilizing: *ANGPT1*–*Tie2* signaling maintains quiescent, mature vessels and supports angiogenic remodeling in the presence of *VEGF*.	[41]
*ANGPT2*	Context-dependent ligand of Tie2 that can antagonize or weakly activate Tie2, promoting endothelial activation, permeability, and vascular remodeling.	Pro-angiogenic but destabilizing: upregulated at sites of vascular remodeling; loosens endothelial–pericyte contacts, making vessels more responsive to *VEGF* and facilitating sprouting or regression depending on *VEGF* levels.	[42]
*ABCC4*	ATP-binding cassette transporter that exports cyclic nucleotides (cAMP, cGMP), prostaglandins, and certain drugs across the plasma membrane.	Indirect modulator: by controlling extracellular prostaglandins and cyclic nucleotides, *ABCC4* can influence endothelial proliferation, migration, and barrier function, processes that underpin angiogenesis.	[43]
*ACSS3*	Mitochondrial acyl-CoA synthetase that converts short-chain fatty acids into acyl-CoA, contributing to cellular energy and lipid metabolism.	Indirect metabolic support: altered *ACSS3* activity can reshape cellular acetyl-CoA pools and bioenergetics, potentially affecting endothelial and stromal cell proliferation and thus angiogenic capacity in metabolic and tumor contexts.	[44]
*ANKRD22*	Ankyrin repeat–containing protein implicated in cell proliferation and inflammatory or cancer-related signaling; exact biochemical role remains incompletely characterized.	Indirect association: differential *ANKRD22* expression has been reported in vascular/angiogenic transcriptomic signatures, suggesting a modulatory role in endothelial or perivascular cell behavior during angiogenesis.	[45]
*E2F7*	Atypical *E2F* transcription factor that represses or modulates genes involved in cell-cycle progression and DNA damage response.	Transcriptional control: *E2F* family members regulate endothelial proliferation and expression of angiogenic factors; *E2F7* can shape angiogenesis by modulating cell-cycle genes and stress responses in endothelial and tumor cells.	[46]
*NRP2*	Co-receptor for *VEGF* family ligands and class 3 semaphorins that modulates guidance, migration, and survival signaling.	Pro-angiogenic co-receptor: enhances *VEGF*-C/*VEGF*-A signaling in endothelial cells, promotes sprouting, lymphangiogenesis, and contributes to pathological tumor vascularization.	[47]
*RGS4*	Regulator of G-protein signaling that accelerates GTP hydrolysis on Gα subunits, turning off GPCR signaling pathways.	Negative/finetuning role: by dampening GPCR signals (e.g., chemokine, thrombin, S1P receptors) in endothelial and mural cells, *RGS4* can modulate migration, vascular tone, and thus the angiogenic response.	[48]
*RYR2*	Intracellular ryanodine receptor Ca^2+^ release channel predominantly in excitable cells, controlling Ca^2+^ oscillations.	Ca^2+^-dependent modulation: endothelial and perivascular Ca^2+^ dynamics are crucial for NO production, contraction, and migration; *RYR*-mediated Ca^2+^ release can influence angiogenic signaling, especially in cardiovascular tissues.	[49]
*THBS1*	Secreted matricellular glycoprotein (thrombospondin-1) that interacts with integrins, CD36, TGF-β, and extracellular matrix components.	Anti-angiogenic: *THBS1* is a classic endogenous inhibitor of angiogenesis, inducing endothelial apoptosis and inhibiting proliferation/migration, thereby counterbalancing pro-angiogenic signals like *VEGF* and *ANGPTs*.	[50]

## Data Availability

The original contributions presented in this study are included in the article/Supplementary Material. Further inquiries can be directed to the corresponding author.

## Supplementary Materials

The following supporting information can be downloaded at: https://www.mdpi.com/article/10.3390/cells15080677/s1. Table S1: Primer sequences for RT-qPCR validation; Table S2: RNA-seq alignment statistics per pool; Table S3: Gene lists for the top 20 enriched GO biological processes. Figure S1: Protein–protein interaction network of consensus DEGs.

## Abbreviations

*ABCC4*	ATP Binding Cassette Subfamily C Member 4
ACSS3	Acyl-CoA Synthetase Short Chain Family Member 3
*ANGPT1*	Angiopoietin 1
*ANGPT2*	Angiopoietin 2
*ANKRD22*	Ankyrin Repeat Domain 22
ART	Assisted Reproductive Technology
*B2M*	Beta-2-Microglobulin
*BMP4*	Bone Morphogenetic Protein 4
*CALCRL*	Calcitonin Receptor Like
CCs	Cumulus Cells
Ct	Cycle Threshold
DEGs	Differentially Expressed Genes
DESeq2	Differential Expression Sequence 2
*E2F7*	E2F Transcription Factor 7
FDR	False Discovery Rate
*GAPDH*	Glyceraldehyde 3-phosphate dehydrogenase
GCs	Granulosa Cells
*GLUL*	Glutamate-Ammonia Ligase
GO	Gene Ontology
GRCh38	Human Genome build 38
*HIF1*	Hypoxia Inducible Factor 1
HISAT2	Hierarchical Spliced Alignments to Transcriptomes 2
HTSeq	High-Throughput Sequence Analysis
*ICAM1*	Intercellular Adhesion Molecule 1
IQR	Interquartile Range
IVF	In Vitro Fertilization
*LIF*	Leukemia Inhibitory Factor
*NRP2*	Neuropilin 2
*NRXN3*	Neurexin 3
PC1/PC2	Principal Component 1 and 2
PCA	Principal Component Analysis
PCR	Polymerase Chain Reaction
*PGE2*	Prostaglandin E2
qPCR	Quantitative Polymerase Chain Reaction
*RGS4*	Regulator of G Protein Signaling 4
RIN	RNA Integrity Number
RNA-seq	RNA sequencing
RT	Room Temperature
RT-qPCR	Reverse Transcription Quantitative PCR
*RYR2*	Ryanodine Receptor 2
SD	Standard Deviation
STAR	Spliced Transcripts Alignment to a Reference
STRING	Search Tool for the Retrieval of Interacting Genes
*THBS1*	Thrombospondin 1
*Tie*	Tyrosine Kinase with Immunoglobulin and EGF homology Domains 2
*VCAM1*	Vascular cell Adhesion Molecule 1
*VEGF*	Vascular endothelial growth factor
