# Supplementary material for: RNA-Seq Analysis of Human Cumulus Cells Identifies Angiogenic Pathways Associated with Infertility
Source: Cells. 2026 Apr 11;15(8):677. doi: 10.3390/cells15080677 (PMC13114718; doi:10.3390/cells15080677)
Supplement: Supplementary file 1 [file cells-15-00677-s001.zip › cells-4220006-supplementary.pdf]

Gene	Forward 5′→3′	Reverse 5′→3′
**ABCC4**	CATTGAGAGGGTGTCAGAGG	GCCTTGTAGAGTTGGGGTCT
**ACSS3**	ACGATGTGATGTAGAGACCCTG	GACGCTTTTTCCTGCTTGCC
**ANGPT1**	TGGGACAGCAGGAAAACAGAG	GCCACAAGCATCAAACCACC
**ANGPT2**	TGGGAAGGGAATGAGGCTTAC	TCCTGGTTGGCTGATGCTG
**ANKRD22**	CAAAGACAAAGCAGAATGAGGC	GCAGAGGGATAAGAGACTGGTT
**E2F7**	CAGTCTTGGTGTGGAAAGGAGA	CTCAGGGTTTTTGGCAGGC
**NRP2**	CCCCGAACCCAACCAGAAGA	CAGTGTTTGCCCAGGAGGTC
**RGS4**	ACATCGGCTAGGTTTCCTGCT	TGACTTCCTCTTGGCTCACTC
**RYR2**	TGAAAGCATCAAACGCAGCA	CAATGAATGTGAGCAGCCCG
**THBS1**	CCAACCGCATTCCAGAGTC	GTCATCAGGCACAGGGGG
**B2M**	GGCTATCCAGCGTACTCCAAA	CGGATGGATGAAACCCAGACA
**GAPDH**	CAAATTCCATGGCACCGTCA	GACTCCACGACGTACTCAGC

Supplementary Table S1. Primer sequences for RT-qPCR validation. Forward and reverse primer sequences (5′→3′) for the ten target genes and two reference genes (B2M, GAPDH) used in this study.

Pool	Group	Nº of readings	Pipeline 1	Pipeline 2	Pipeline 3
				% Aligned	
Pool_D1	Donor	71295392	98.94	88.9	96.69
Pool_D2	Donor	79865035	99.22	90.3	97.08
Pool_PM1	Patient	66047275	99.15	88.9	96.81
Pool_PM2	Patient	59152441	99.02	90	96.78

Supplementary Table S2. RNA-seq alignment statistics per pool. Total reads and percentage of reads aligned to the GRCh38.p13 reference genome for each of the four pooled samples (Pool_D1, Pool_D2, Pool_PM1, Pool_PM2) across the three bioinformatic pipelines.

Gene	Forward 5′→3′	Reverse 5′→3′
**Positive regulation of cell motility (GO:2000147)**	ANGPT1, CFAP20, CXCR4, EPB41L4B, HAS2, LYVE1, MMP14, PROX1, WISP1	ADAMTS1, BMP2, BMP4, CCL3, CCL4, CHD13, EDN2, ETS1, GLIPR2, ICAM1, IL1B, IL6, NRP2, PLAU, PPAP2B, PRKCA, S1PR1, SEMA3B, THBS1, ZC3H12A
**Tube morphogenesis (GO:0035239)**	ANGPT1, CALCRL, CITED1, EFNB2, GJA5, GLI3, GLUL, HAS2, MAPK14, MMP14, PROX1	ADAMTS16, ADM, BMP2, BMP4, CDH13, E2F7, EDN2, JUNB, MTSS1, MYC, NFIB, NINJ1, NRP2, NRXN3, PRKCA, RYR2, S1PR1, SDC4, THBS1, ZC3H12A, ZMIZ1
**Regulation of cell adhesion (GO:0030155)**	ANGPT1, CXCR4, EFNB2, EPB41L4B, GLI3, HAS2, HLA-DQB2, MAPK14, MMP14, PCK1, PRKAA1	BMP2, BMP4, CDH13, EDIL3, ETS1, FRMD5, FSTL3, HLA-DRB1, HLA-DRB5, IL1B, IL6, IRF1, LIF, NFKBIZ, PLAU, PPAP2B, PRKCA, S1PR1, SDC4, THBS1, VCAM1, ZC3H12A, ZMIZ1
**Positive regulation of cell migration (GO:0030335)**	ANGPT1, CXCR4, EPB41L4B, HAS2, LYVE1, MMP14, PROX1, WISP1	ADAMTS1, BMP2, BMP4, CCL3, CCL4, CHD13, EDN2, ETS1, GLIPR2, ICAM1, IL1B, IL6, NRP2, PLAU, PPAP2B, PRKCA, S1PR1, SEMA3B, THBS1, ZC3H12A
**Positive regulation of locomotion (GO:0040017)**	ANGPT1, CFAP20, CXCR4, EPB41L4B, HAS2, LYVE1, MMP14, PROX1, WISP1	ADAMTS1, BMP2, BMP4, CCL3, CCL4, CHD13, EDN2, ETS1, GLIPR2, ICAM1, IL1B, IL6, NRP2, PLAU, PPAP2B, PRKCA, S1PR1, SEMA3B, THBS1, ZC3H12A
**Vasculature development (GO:0001944)**	ANGPT1, CALCRL, CITED1, EFNB2, GJA5, GLI3, GLUL, HAS2, MAPK14, MMP14, PROX1	ADM, BMP2, BMP4, CDH13, E2F7, EDN2, JUNB, LIF, NINJ1, NRP2, NRXN3, PPAP2B, PRKCA, S1PR1, THBS1, ZCEH12A, ZMIZ1
**Regulation of locomotion (GO:0040012)**	ANGPT1, ARHGAP18, CFAP20, CXCR4, EFNB2, EPB41L4B, GLUL, HAS2, LYVE1, MMP14, MPP1, PLCB1, PROX1, SSX2IP, WISP1	ADAMTS1, BMP2, BMP4, CCL3, CCL4, CD200, CDH13, EDN2, ETS1, FRMD5, GLIPR2, ICAM1, IL1B, IL6, NINJ1, NRP2, PLAU, PPAP2B, PRKCA, S1PR1, SDC4, SEMA3B, THBS1, ZC3H12A
**Regulation of cell motility (GO:2000145)**	ANGPT1, ARHGAP18, CFAP20, CXCR4, EPB41L4B, GLUL, HAS2, LYVE1, MMP14, MPP1, PLCB1, PROX1, SSX2IP, WISP1	ADAMTS1, BMP2, BMP4, CCL3, CCL4, CD200, CDH13, EDN2, ETS1, FRMD5, GLIPR2, ICAM1, IL1B, IL6, NINJ1, NRP2, PLAU, PPAP2B, PRKCA, S1PR1, SDC4, SEMA3B, THBS1, ZC3H12A
**Blood vessel morphogenesis (GO:0048514)**	ANGPT1, CALCRL, CITED1, EFNB2, GJA5, GLI3, GLUL, HAS2, MAPK14, MMP14, PROX1	ADM, BMP4, CDH13, E2F7, EDN2, JUNB, NINJ1, NRP2, NRXN3, PRKCA, S1PR1, THBS1, ZC3H12A, ZMIZ1
**Tube development (GO:0035295)**	ANGPT1, CALCRL, CCKBR, CITED1, EFNB2, GJA5, GLI3, GLUL, HAS2, HDC, MAPK14, MMP14, PROX1,	ADAMTS16, ADM, BMP2, BMP4, CDH13, E2F7, EDN2, JUNB, MTSS1, MYC, NFIB, NINJ1, NRP2, NRXN3, PRKCA, RYR2, S1PR1, SDC4, THBS1, ZC3H12A, ZMIZ1
**Blood vessel development (GO:0001568)**	ANGPT1, CALCRL, CITED1, EFNB2, GJA5, GLI3, GLUL, HAS2, MAPK14, MMP14, PROX1	ADM, BMP4, CDH13, E2F7, EDN2, JUNB, NINJ1, NRP2, NRXN3, PPAP2B, PRKCA, S1PR1, THBS1, ZC3H12A, ZMIZ1
**Circulatory system development (GO:0072359)**	ANGPT1, CALCRLC CITED1, EFNB2, GJA5, GLI3, GLUL, HAS2, MAPK14, MMP14, PROX1,	ADAMTS1, ADM, BICC1, BMP2, BMP4, CDH13, DNAH11, E2F7, EDN2, HSPB7, INHBA, JUNB, LIF, NINJ1, NRP2, NRXN3, PPAP2B, PRKCA, RYR2, S1PR1, THBS1, VCAM1, ZC3H12A, ZMIZ1

**Regulation of cell migration (GO:0030334)**	ANGPT1, CXCR4, EPB41L4B, GLUL, HAS2, LYVE1, MMP14, MPP1, PLCB1, PROX1, WISP1	ADAMTS1, BMP2, BMP4, CCL3, CCL4, CD200, CDH13, EDN2, ETS1, FRMD5, GLIPR2, ICAM1, IL1B, IL6, NINJ1, NRP2, PLAU, PPAP2B, PRKCA, S1PR1, SDC4, SEMA3B, THBS1, ZC3H12A
**Response to cytokine (GO:0034097)**	ABCA1, ALAD, CITED1, CXCR4, GHR, HAS2, MAPK14, PLCB1	BIRC3, CCL3, CCL4, CD200, CDC42EP2, EDN2, GBP2, ICAM1, IL1B, IL6, INHBA, IRF1, MYC, NFKB1, NFKB2, NFKBIA, NFKBIZ, NRP2, PRKCA, THBS1, TRAF1, TUBA1A, VCAM1, ZC3H12A
**Positive regulation of cell adhesion (GO:0045785)**	ANGPT1, EFNB2, EPB41L4B, GLI3, HAS2, HLA-DQB2, PCK1, PRKAA1	CDH13, EDIL3, ETS1, FRMD5, FSTL3, HLA-DRB1, HLA-DRB5, IL1B, IL6, LIF, NFKBIZ, PPAP2B, PRKCA, SDC4, VCAM1, ZMIZ1
**Response to peptide (GO:1901652)**	ABCA1, ALAD, CITED1, CXCR4, GHR, HAS2, MAPK14, PLCB1	BIRC3, CCL3, CCL4, CD200, CDC42EP2, EDN2, GBP2, ICAM1, IL1B, IL6, INHBA, IRF1, MYC, NFKB1, NFKB2, NFKBIA, NFKBIZ, NRP2, PRKCA, THBS1, TRAF1, TUBA1A, VCAM1, ZC3H12A
**Response to bacterium (GO:0009617)**	ABCA1, ALAD, CITED1, EFNB2, HDC, KLHL6, MAPK14, PCK1, SLC9A9	ADM, BCL3, BMP2, CD200, CXCL2, GBP2, HLA-DRB1, HMCN1, IL1B, IL6, NFIB, NFKB1, NFKB2, NFKBIA, NFKBIZ, PTGFR, RNASE1, VCAM1, ZC3H12A
**Response to lipid (GO:0033993)**	ABCA1, ALAD, CITED1, EFNB2, GHR, MAPK14, MMP15, PCK1, PLCB1, PRKAA1, TRIM16	CA9, CCL3, CD200, FABP3, GBP2, IL1B, IL6, INHBA, JUNB, NFKB1, NFKB2, NFKBIA, NFKBIZ, PRKCA, PTGFR, THBS1, VCAM1, ZC3H12A, ZMIZ1
**Positive regulation of cell differentiation (GO:0045597)**	CXCR4, EFNB2, GHR, GLI3, GLUL, ID4, MAPK14, MMD, MMP14, PCK1, PLCB1, PROX1, TRIM16, WISP1	ADM, BMP2, BMP4, ETS1, GLIPR2, HLA-DRB1, IL1B, IL6, INHBA, JUNB, LIF, NFKB1, NFKBIZ, PRKCA, ZC3H12A, ZMIZ1
**Angiogenesis (GO:0001525)**	ANGPT1, CALCRL, EFNB2, GJA5, GLUL, MAPL14, MMP14	BMP4, CDH13, E2F7, EDN2, NINJ1, NRP2, NRXN3, PRKCA, S1PR1, THBS1, ZC3H12A

Supplementary Table S3. Gene lists for the top 20 enriched GO biological processes. For each significantly enriched term, the associated upregulated and downregulated genes from the consensus DEG set are listed. Key angiogenesis-related genes are highlighted.

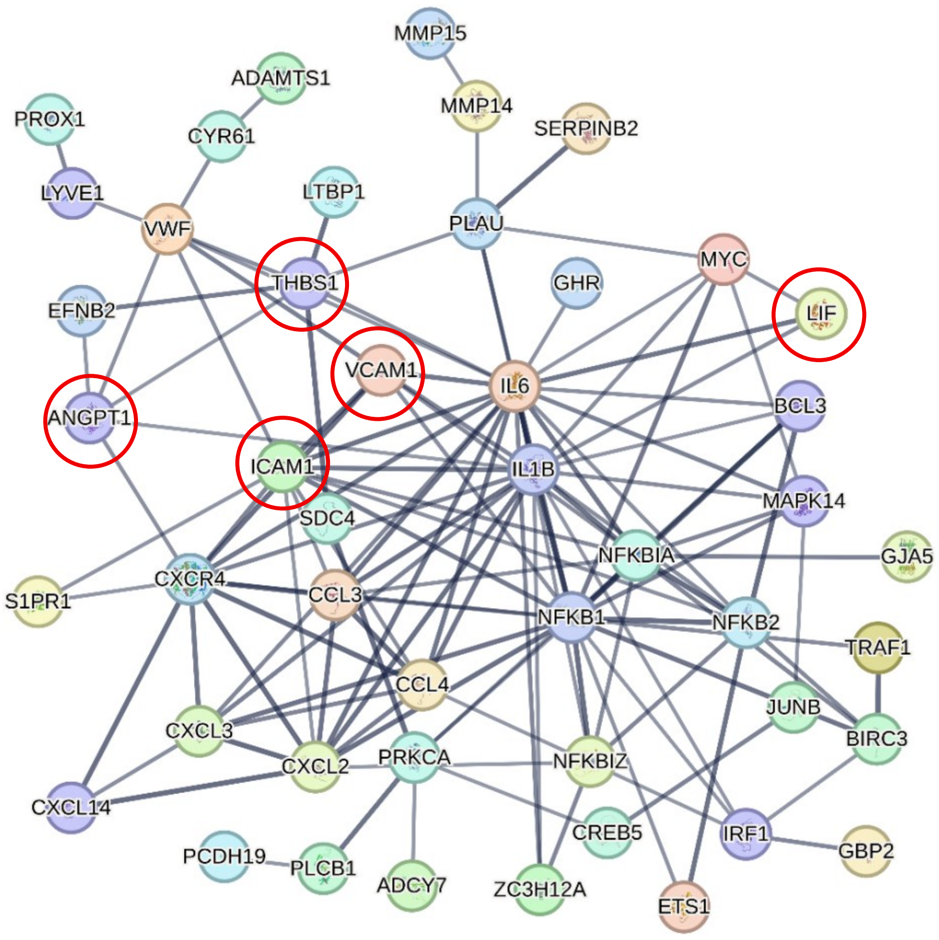

Supplementary Figure S1. Protein-protein interaction network of consensus DEGs. STRING analysis of the 234 consensus DEGs, showing interactions with a confidence threshold >0.7. Key angiogenesis-related genes (ANGPT1, THBS1, BMP4, GLUL) and endothelial factors (VCAM1, ICAM1, LIF) form central hubs in the network.
